# Highly Sensitive Trimetazidine Determination Using Composite Yttria-Stabilized Zirconia Doped with Titanium Oxide–Carbon Black Biosensor

**DOI:** 10.3390/ma17225556

**Published:** 2024-11-14

**Authors:** Małgorzata Suchanek, Agata Krakowska, Beata Paczosa-Bator, Robert Piech

**Affiliations:** 1 Department of Analytical Chemistry and Biochemistry, Faculty of Materials Science and Ceramics, AGH University of Krakow, Al. A. Mickiewicza, 30-059 Krakow, Poland; agata.krakowska@uj.edu.pl (A.K.); paczosa@agh.edu.pl (B.P.-B.); 2 Department of Inorganic Chemistry and Pharmaceutical Analytics, Faculty of Pharmacy, Jagiellonian University Medical College, 9 Medyczna Street, 30-688 Kraków, Poland

**Keywords:** trimetazidine, yttria-stabilized zirconia doped with titanium dioxide, voltammetry, pharmaceutical formulation, urine sample, blood plasma sample, digestive juice

## Abstract

A novel composite voltammetric biosensor has been developed for the first time, utilizing a glassy carbon electrode modified with yttria-stabilized zirconia doped with titanium dioxide and carbon black (YSZTiO_2_-CB/GCE), specifically designed for the detection of trimetazidine (TMZ). The measurement conditions, including both the supporting electrolyte and instrumental settings, were optimized to enhance performance. In the concentration range of 0.5 to 7 µM, it is not necessary to use preconcentration time for the determination of TMZ. The limit of detection (for 60 s of preconcentration time) was equal to 5.5 nM (1.87 ng mL^−1^), outperforming other voltammetric methods in terms of sensitivity. The reproducibility of the trimetazidine signal (with a concentration of 0.05 µM) exhibited a relative standard deviation (RSD) of 3.3% over 10 measurements. Additionally, our biosensor is characterized by excellent stability, ease of use, and straightforward preparation. The proposed biosensor and method have proven effective in analyzing TMZ in a variety of matrices, including urine, blood plasma, pharmaceutical formulations, as well as gastric and intestinal fluids, yielding recovery rates ranging from 97.7 to 102.3%.

## 1. Introduction

Trimetazidine (TMZ) is an organic chemical compound known as 1-(2,3,4-trimethoxybenzyl)piperazine and is a drug commonly used to treat angina pectoris, a condition characterized by chest pain due to reduced blood flow to the heart. It is also employed in the management of ischemia-related conditions affecting neurosensory tissues, such as Meniere’s disease. TMZ’s efficacy in treating angina is comparable to propranolol, although it differs by not lowering the cardiac rate–pressure product or coronary blood flow [1]. It plays a role in regulating ionic exchanges and extracellular balance, correcting the ion flow of ion disturbances induced by ischemia, and preventing cell swelling due to oxygen deprivation [2]. A cytoprotective agent, TMZ acts by inhibiting fatty acid β-oxidation in hypoxic heart cells, promoting glucose oxidation instead. This helps preserve intracellular ATP levels, ensuring proper sodium–potassium pump function and maintaining cellular stability without significant hemodynamic effects. TMZ achieved peak serum concentration approximately five hours post-administration, reaching a steady state within 60 h. It is primarily through the urine in unchanged form, with a half-life of around seven hours [3]. While neutral TMZ shows limited solubility in aqueous solutions, its dihydrochloride salt form is readily soluble in water and somewhat soluble in alcohol [4]. Studies have shown that TMZ improves psychomotor performance, reduces fatigue, and enhances cardiovascular function in healthy individuals exposed to high altitudes [5,6]. Therefore, the TZM was classified as a performance-enhancing substance by the World Anti-Doping Agency (WADA) in 2014, adding it to their list of prohibited substances [7]. Consequently, it is important to identify a suitable method for detecting and determining trimetazidine at the simple, sensitive, cost-effective and selective analytical methods with low detection limits suited to human biofluids and medicinal products.

Several methods for determining trimetazidine have been documented, including UV–visible spectrophotometry [8,9,10], gas chromatography–mass spectrometry [11,12], liquid chromatography coupled with mass spectrometry (LC–MS) [13], HPLC [2,14,15], chemiluminescence [16], flow injection analysis [17], spectrofluorimetric [18], and capillary electrophoresis with amperometric detection [19]. Electroanalytical methods for the analysis of pharmaceuticals and biological samples have gained momentum due to advancements in voltammetric techniques and the development of sensitive electrode materials. Voltammetric measurements are conducted on various types of working electrodes to detect both inorganic and organic substances [20,21,22,23]. The glassy carbon electrode (GCE) with various surface modifiers is the most frequently used electrode in voltammetry [24,25,26]. Other electrodes include the hanging mercury drop electrode (HMDE), carbon paste electrode, boron-doped diamond electrode (BGDE) [27], and graphite electrode (GRE) [28] used in voltammetric determination. Various surface modification strategies, such as the use of conductive polymers [26,29], metal nanoparticles [25,30] or yttria-stabilized zirconia (YSZ) [31,32], have been applied to improve electrode performance, with some studies modifying YSZ using neodymium [33].

Pure zirconia, known for its excellent resistance to fracture and chemicals, is commonly used as a thermal barrier coating and is a substitute for diamond in various applications. Zirconia exists in three crystal forms: monoclinic, tetragonal, and cubic, depending on the temperature. The high-temperature structure of zirconia oxide exhibits the best properties of chemical and fracture toughness. The addition of various oxides causes the synthesis of such a structure at room temperature. The addition of different percentages of yttria to the structure allows for the observation of tetragonal and monoclinic phases during synthesis at lower temperature [34]. The yttrium oxide–doped zirconium oxide solid solution (YSZ) can be further modified by incorporating rare earth oxides such as lanthanum, samarium, gadolinium, or neodymium [35,36,37,38]. For the first time, YSZ doped with TiO_2_ has been synthesized and used for modification of a glassy carbon electrode.

The focus of this research is to present a novel analytical application of YSZTiO_2_-CB composite-modified glassy carbon electrodes for determining trimetazidine in pharmaceutical products, urine, blood plasma, and artificial digestive juice. By combining carbon nanomaterials with YSZTiO_2_ nanopowder, the surface area of the working electrode is significantly increased. This biosensor delivers superior detection limits compared to other voltammetric methods for trimetazidine determination. Its effectiveness was validated by measuring TMZ in pharmaceutical products, as well as in urine and blood plasma samples. Additionally, the proposed sensor was used to measure TMZ in digestive juices, following an experiment in which TMZ was extracted into an artificial digestive system. The results confirm the sensor’s suitability for TMZ detection across a range of samples.

## 2. Materials and Methods

### 2.1. Apparatus

A multi-tasking instrument (model M161) equipped with appropriate electrode platform type M164 (producer mtm-anko, Krakow, Poland) coupled with dedicated EAQt software (http://home.agh.edu.pl/~kca/home/, accessed on 1 January 2024) was used to control the measuring equipment and record measurement data. The cyclic voltammetry measurements were realized using the device VersaSTAT4 (company Ametek Inc., Berwyn, PA, USA). A standard 20 mL volumetric cell setup was employed, featuring a double junction incorporating ceramic sinters Ag/AgCl/KCl (3M) as the standard reference electrode. A platinum rod (diameter 0.5 mm) was used as the supporting electrode, and a 3 mm diameter glassy carbon electrode (GCE) (producer Mineral, Łomianki-Sadowa, Poland) was used as the substrate electrode. This electrode was subjected to modification with a combination of well-defined carbon black (CB) and yttria-stabilized zirconia nanoparticles, along with titanium oxide (YSZTiO_2_-CB/GCE), to serve as the detector working electrode. The obtained curves were imagined and processed using Matlab2019a. The buffer solution pH was determined using the Elmetron CX-705 multi-instrument (producer Elmetron, Zabrze, Poland).

Electrical impedance spectroscopy (EIS) measurements were taken using the previously mentioned VersaSTAT4 instrument. The measurements were made on a standard three-electrode system with the double-junction Ag/AgCl/KCl (3 M) as a reference electrode, a platinum spiral as the auxiliary electrode, and GC electrodes modified with YSZTiO_2_, carbon black and YSZTiO_2_—carbon black as a working electrode. All data were processed using the ZSimWin 3.60 software.

The specific surface area and whole nanopore volume of the obtained YSZTiO_2_ nanopowder were analyzed using N_2_ adsorption techniques following the Brunauer–Emmett–Teller (BET) method (ASAP 2010, Micromeritics, Norcross, GA, USA). The nanostructure and surface morphology of YSZTiO_2_, carbon black, and the YSZTiO_2_–carbon black layer were examined using a scanning electron microscope (SEM) (Apreo 2S Scanning Electron Microscope, Thermo Fisher Scientific, Hillsboro, OR, USA) with EDX analysis at an accelerating voltage of 10 kV and magnifications up to 50,000.

### 2.2. Chemicals and Glassware

All reagents used were of high purity according to the manufacturer’s specifications. The standard solution of trimetazidine (TMZ) (0.01 M) was purchased from Sigma-Aldrich (St. Louis, MO, USA) and was dissolved in highly pure water. The solution was shielded from light and kept refrigerated between measurements. Fresh solutions with 0.001 M and 0.0001 M of TMZ concentrations were prepared every day by dissolving the appropriate amount of the compound in double-distilled water. The other reagents were purchased as follows: carbon black CAT from 3D-nano (Poland), N,N-dimethyloformamide, zirconium oxychloride octahydrate (ZrOCl·8H_2_O) as a powder, yttrium oxide (Y_2_O_3_), and titanium oxide were obtained from Sigma-Aldrich. Freeze-dried urine was obtained from Medichem (Steinenbronn, Germany), while human plasma was obtained from Biowest (Naualle, France). The selection of interferents was based on their presence in pharmaceutical products and biological samples. A similar criterion for the selection of interferents was used in our previous studies [33,39].

### 2.3. Procedures

#### 2.3.1. YSZ Nanopowder Preparation

An amount of 3 mol% yttria-stabilized zirconia (YSZ) was synthesized using the precipitated method. The solution containing ZrO_2_ and Y_2_O_3_ in a ratio of 97:3 mol% was subjected to precipitation with an ammonia–water solution. The resulting gel was thoroughly washed with double-distilled water to remove residual ammonium chloride and nitrate salts. Following this, the co-precipitated gel was dried at 240 °C for 8 h. The dried YSZ powder was then blended with titanium oxide at a 95:5 weight ratio and ground in a rotary–vibratory mill using isopropyl alcohol as the suspension medium for 6 h at a speed of 350 rpm. Zirconium balls (1.5 mm in diameter) were used as the grinding medium. After milling, the nanopowder was heated in a furnace at 850 °C, with heating and cooling rates set at 100 °C h^−1^. The resulting nanopowder was subsequently crushed in an agate mortar and used to modify the glassy carbon electrode.

#### 2.3.2. Preparation of the Modified Electrode

To create the modifier suspension, 10 mg of carbon black and 5 mg of YSZTiO_2_ nanopowder were accurately weighed and quantitatively transferred into a 10 mL volumetric flask. The amount of YSZTiO_2_ nanopowder was optimized, dispersed, and sonicated in a suitable amount of N,N-dimethyloformamide for around 15 min.

Following this, the GC electrode was coated with the prepared YSZTiO_2_-CB composite. Prior to modification, the surface of the GCE was polished using a 0.3 µm Al_2_O_3_ slurry and thoroughly rinsed with a double-distilled water stream. The electrode was then submerged in acetone and sonicated for 3 min. After cleaning, 10 µL of the modifier suspension was applied onto the polished GCE surface. The modified electrode was left to dry under a glass cover for 8 h at room temperature. Once prepared, the electrode remained usable for up to 1 month.

#### 2.3.3. Sample Preparation

**Figure 1:**
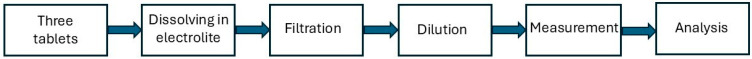
A scheme presenting the tablet preparation procedure for voltammetric analysis.

Pharmaceutical products (containing 35 mg trimetazidine dihydrochloride per tablet) were purchased from the local pharmacy and were prepared according to the standard procedure and to the scheme presented in Figure 1. The urine (Medidrug, Barcelona, Spain) and human plasma samples (Biowest, Naualle, France) were prepared following the manufacturer’s instructions. Detailed descriptions of the real sample preparation methods can be found in our previous publications [33,39,40,41,42].

#### 2.3.4. The Extraction of Trimetazidine into Artificial Digestive Juice

In the presented work, the degree of extraction of the active substance contained in the drug into the digestive system was determined. For this purpose, the analyzed Setal MR tablets were extracted into artificial digestive juices (gastric and intestinal). Extractions were carried out for the minimum dose (1 tablet = 35 mg) and the maximum daily dose (2 tablets = 70 mg) recommended by the manufacturer. Initially, the tablets were moistened with 3 mL of an artificial saliva solution prepared according to Arvidson’s model (composition: Na_2_HPO_4_, KHCO_3_, KH_2_PO_4_, C_6_H_8_O_7_, MgCl_2_, CaCl_2,_ and NaCl (Sigma-Aldrich) [43]. Then, 10 mL of artificial gastric juice was added to the samples (composition: NaCl (Sigma-Aldrich), HCl Suprapur^®^ (Merck, Darmstadt, Germany), and pepsin (POL-AURA, Olsztyn, Poland) (according to the Polish Pharmacopoeia). The samples were incubated in artificial gastric juice for 60 and 120 min in a water bath with a shaker, with the temperature maintained at 36 ± 2 °C. Then, the tablets were separated, and the filtrate was filtered through membrane filters (pore diameter 0.22 μm). An amount of 10 mL of artificial intestinal juice (composition: pancreatic extract (Merck, Darmstadt, Germany), bile salts (BTL, Łódź, Poland), and NaHCO_3_ (Sigma-Aldrich, Poznań, Poland)) was added to the obtained separated and pre-digested material and incubated again for 150 min. Then, the previous steps were repeated. To obtain artificial digestive juices, quadruple-distilled water with a conductivity of less than 2 µS was used, which was obtained using a quartz distiller. All the above steps for each sample were performed in three independent repetitions. The filtrates obtained in the manner described above were analyzed.

#### 2.3.5. Measurements Procedure

The differential pulse voltammetry (DPV) technique was used to quantitatively trimetazidine determination. A specified volume of the TMZ standard solution was added to a 20 mL voltammetric cell, after which acetate buffer was diluted to a final volume of 10 mL (0.05 M concentration) at pH 4.5, and the measurements were then performed. The cyclic voltammetry technique was applied to characterize the properties of the YSZTiO_2_-CB/GCE layer. Unless otherwise provided, the DP voltammograms were registered as follows: sampling and waiting time t_p_ = t_s_ = 20 ms, step potential E_s_ = 5 mV, pulse amplitude height dE = 40 mV, recording scan potential in the range of 400 to 1200 mV. To ensure high repeatability of the TMZ signal, it was necessary to implement a 15 s rest period between each voltammogram recording.

## 3. Results and Discussion

### 3.1. Examination of Electrode Characteristics

**Figure 2:**
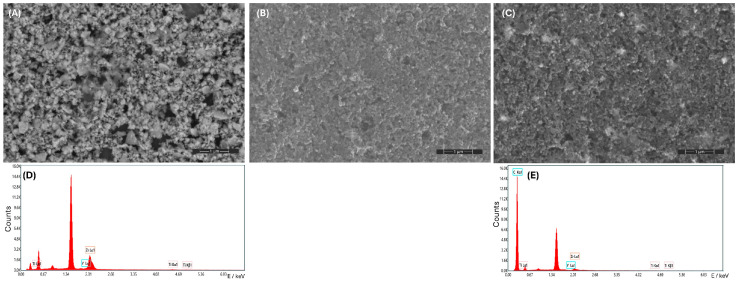
Scanning electron microscope (SEM) micrographs of (**A**) YSZTiO_2_, (**B**) carbon black, (**C**) YSZTiO_2_ with carbon black with energy dispersive X-ray (EDX) spectra of (**D**) YSZTiO_2,_ and (**E**) YSZTiO_2_ with carbon black. Also shown are an accelerating voltage of 10 kV and a magnification of 50,000.

The morphology of YSZTiO_2_, CB, and YSZTiO_2_-CB layers obtained using SEM microscopy is presented in Figure 2. As can be seen, the particles observed in Figure 2A were a size smaller than 1 µm. The YSZTiO_2_ contains small particles of cubic shapes. The carbon black contains nanometric grains (Figure 2B). In Figure 2C, the YSZTiO_2_ with carbon black is observed. Small single YSZTiO_2_ grains are shown. The EDX analysis of the chemical composition verified the modification of the YSZ structure with the addition of TiO_2_ (Figure 2D,E). The titanium content incorporated into the YSZ framework, calculated as the average atomic concentration across multiple surface points, was 5.95% for YSZTiO_2_ and 1.10% for YSZTiO_2_-CB. BET analysis revealed that the specific surface area of the YSZTiO_2_ nanopowder was 31.12 m^2^ g^−1^, with a pore volume of 0.138 cm^3^ g^−1^ and an average pore diameter of 1.13 nm.

**Figure 3:**
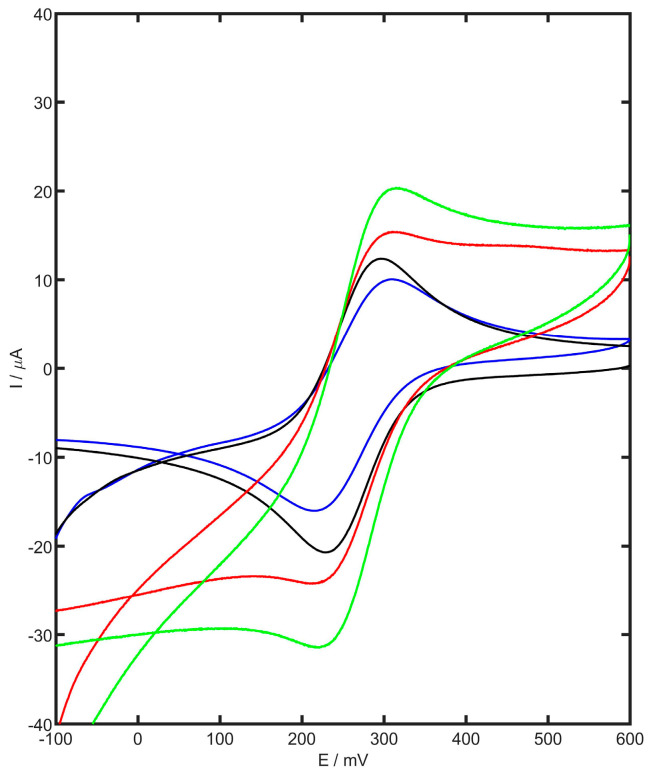
Cyclic voltammetry of 1 mM K_3_[Fe(CN)_6_] in 1 M KCl for GC electrode (blue), YSZTiO_2_/GC electrode (black), CB/GC electrode (red), and YSZTiO_2_-CB/GC electrode (green).

The modification of the GCE surface using carbon black and YSZTiO_2_ was analyzed using electrochemical impedance spectroscopy (EIS) and cyclic voltammetry (CV). Cyclic voltammograms were recorded for the unmodified glassy carbon electrode, GC modified with YSZTiO_2_, GC modified with carbon black, and GC modified with both carbon black and YSZTiO_2_ in a 1 mM of K_3_[Fe(CN)_6_] in 1 M KCl. The resulting voltammograms for each electrode, obtained at a scan rate of 100 mV s^−1,^ are shown in Figure 3. The electrochemically active surface area of each electrode was determined using the Randles–Ševčik equation [44]:

(1) $$I_{p}=\left(2.69\times 10^{5}\right)n^{3/2}A_{el}D^{1/2}v^{1/2}c$$

where $I_{p}$ is a peak current, *A_el_* is the electrochemically activated surface area of the electrode, and *v* is the scan rate (*v* = 100 mV s^−1^), $D=7.6\times 10^{-6}$ cm^2^ s^−1^.

As shown in Table 1, the bare GCE exhibited a well-defined voltammogram with symmetric anodic and cathodic peaks and a peak separation ΔE of 68 mV. When the electrode was modified with YSZTiO_2_, the anodic and cathodic currents increased to 15.12 µA and 15.73 µA, respectively, compared to the unmodified GCE (11.59 µA and 12.07 µA). A further increase in peak currents was observed with the YSZTiO_2_-CB/GCE, where the anodic and cathodic current values reached 20.68 µA and 20.47 µA, respectively. The voltammogram of YSZTiO_2_-CB/GCE displayed a near-theoretical peak separation ΔE of 60 mV. The calculated electrochemically active surface area of YSZTiO_2_-CB/GCE was 21% larger than CB/GCE and 76% larger than the bare GCE. The significant increase in active surface area, along with enhanced electron transfer, improves the efficiency of the electrode reaction, leading to higher recorded currents and greater sensitivity.

**Figure 4:**
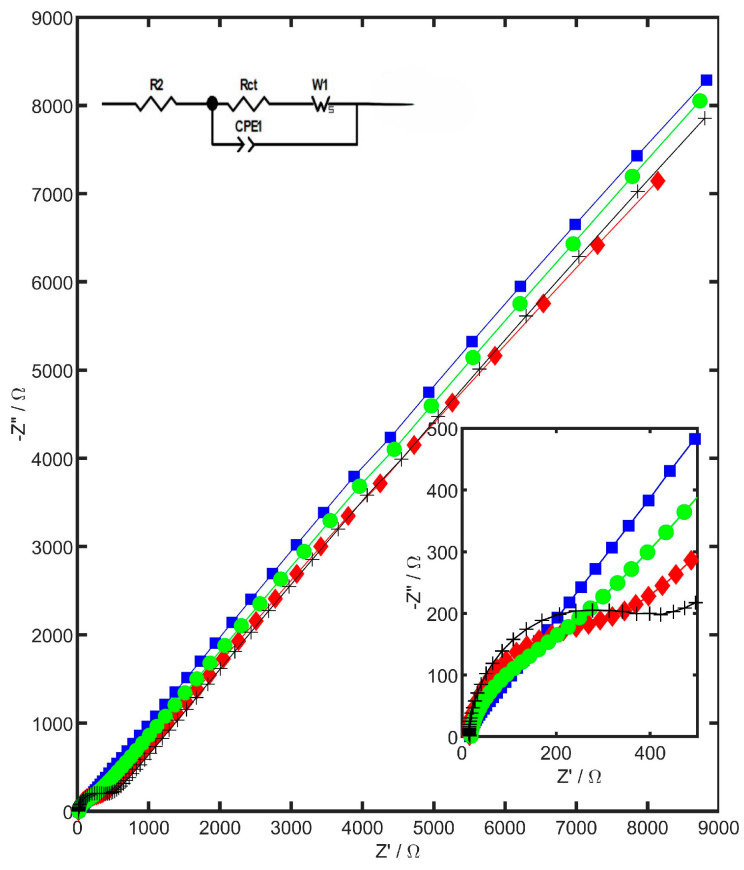
Nyquist plots recorded for 1 mM K_3_[Fe(CN)_6_] in 1 M KCl solution for GC electrode (blue), YSZTiO_2_/GC electrode (black), CB/GC electrode (red), and YSZTiO_2_-CB/GC electrode (green).

The electrical impedance spectroscopy (EIS) measurements were carried out using 1 mM of K_3_[Fe(CN)_6_] in 1 M KCl, with sinusoidal signals applied over a frequency range from 100 kHz to 25 mHz. A small excitation amplitude of 10 mV peak-to-peak was applied at the formal potential of K_3_[Fe(CN)_6_]. To evaluate the influence of specific nanocomponents on the metrological characteristics of the working electrode, Nyquist plots were recorded for bare GCE, YSZTiO_2_/GCE, CB/GCE, and YSZTiO_2_-CB/GCE, as shown in Figure 4.

The obtained EIS spectra enabled the determination of parameters such as charge transfer resistance (*R_ct_*) and effective capacitance (*C_eff_*). The capacitance was calculated using ZSimpWin 3.60 software and the following equation:

(2) $$C_{eff}=CPE^{1/N}\times {\left[1/R_{2}+1/R_{ct}\right]}^{\left(N-1\right)/N}$$

The obtained effective capacitances achieved 0.291, 0.461, 0.625, and 1.126 µF for GCE, the YSZTiO_2_/GC electrode, the CB/GC electrode, and the YSZTiO_2_-CB/GC electrode, respectively. The capacitance increases due to the enhancement of the electrochemical active surface area associated with the modification of the modifier composition. The obtained parameters are presented in Table 2.

The obtained EIS spectra indicate that modifying the GCE surface with YSZTiO_2_ leads to a noticeable increase in charge transfer resistance (*R_ct_*) and a slight rise in capacitance (*C_eff_*), indicating that the applied layer impedes electron transfer due to its low electron conductivity. Introducing carbon black into the YSZTiO_2_ matrix significantly enhances the capacitance and dramatically reduces the *R_ct_* in comparison to YSZTiO_2_-modified GCE, improving the efficiency of the electrode reaction. The YSZTiO_2_-CB/GCE exhibits the most favorable electrical properties, with the lowest *R_ct_* and highest value of capacitance, resulting in well-defined voltammograms and an optimal signal-to-noise (S/N) ratio [45,46].

### 3.2. Effect of Modifier

**Figure 5:**
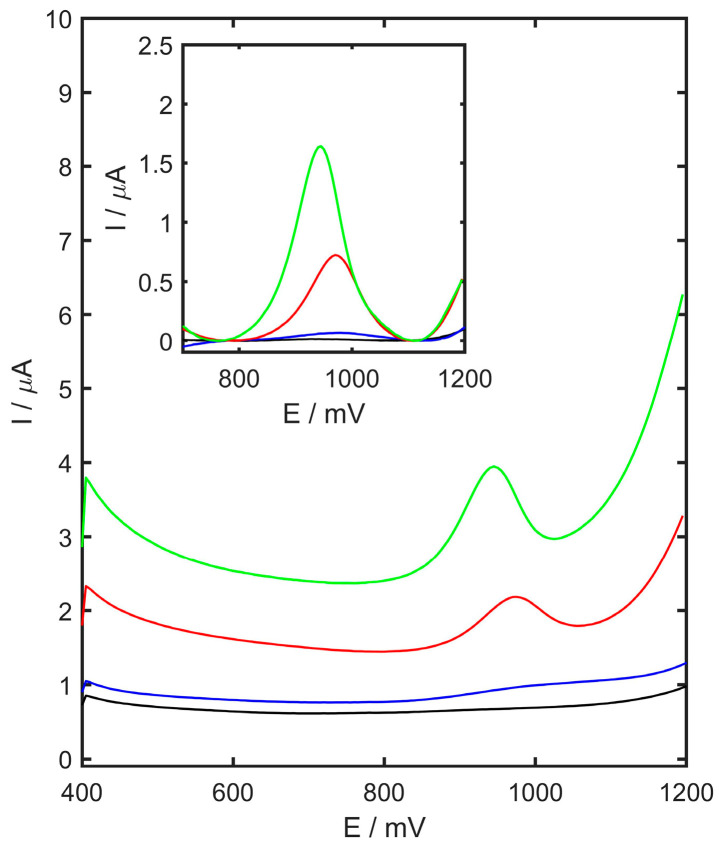
Differential pulse voltammograms recorded for 0.05 M acetate buffer at pH 4.5 (black) onto GCE, and 2 µM TMZ in 0.05 M acetate buffer (pH 4.5) onto GCE (blue), CB/GCE (red), and YSZTiO_2_-CB/GCE (green).

The electrochemical oxidation of TMZ was investigated using differential pulse voltammetry in acetate buffer (pH 4.5) on GCE, CB/GCE, and YSZTiO_2_-CB/GCE with 2 µM of TMZ. A distinct oxidation peak for TMZ was detected on electrodes modified with carbon black and the YSZTiO_2_-carbon black combination. As shown in Figure 5, the peak potential was lower for YSZTiO_2_-CB/GCE compared to CB/GCE (943 mV and 972 mV, respectively), indicating that theYSZTiO_2_-modified electrodes may exhibit catalytic activity. The peak currents recorded for YSZTiO_2_-CB/GCE, CB/GCE, and the unmodified GC were equal to 1.63, 0.72, and 0.08 µA, respectively.

**Figure 6:**
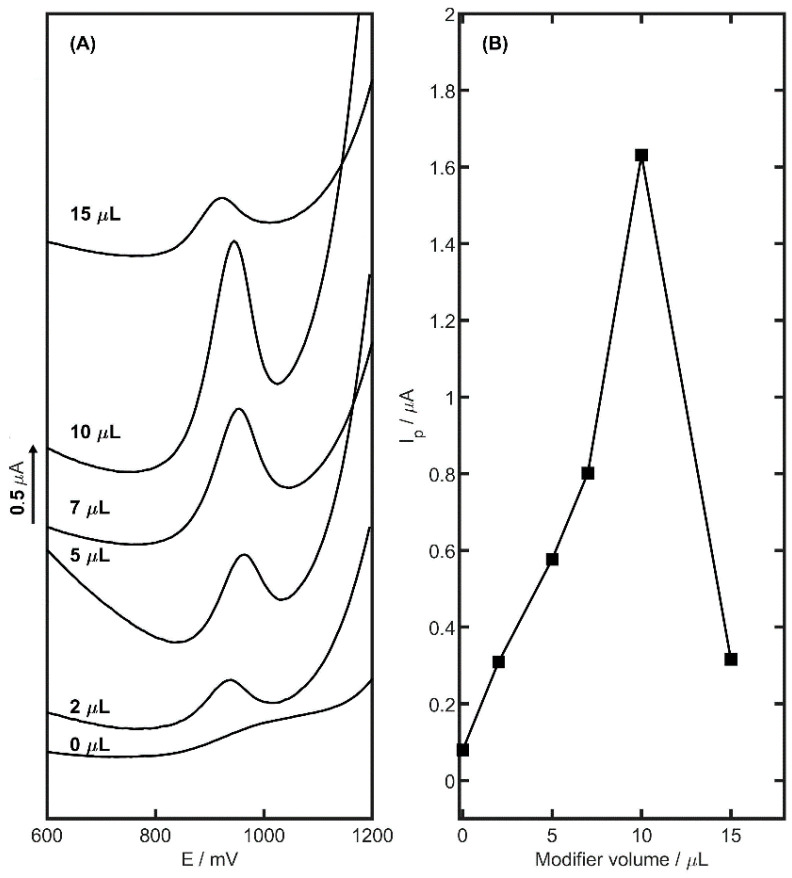
The dependence of TMZ peak current on the volume of YSZTiO_2_-CB modifier on the surface of GCE, DP voltammograms (**A**), and plot (**B**). The electrolyte was 0.05 M acetate buffer with pH 4.5 and 2 µM of TMZ concentration.

The volume of surface modifier applied played a key role in the electrode’s electrochemical performance. Therefore, various volumes of suspension were tested to optimize this parameter. Five GCEs were coated with YSZTiO_2_-CB suspensions in volumes of 2, 5, 7, 10, and 15 µL. The measurements, conducted with 2 µM TMZ in 0.05 M acetate buffer at pH 4.5, are presented in Figure 6. The highest TMZ peak current was observed with a 10 µL suspension, Making it the optimal volume for further experiments. Although smaller modifier volumes produced higher peaks, an increase to 15 µL led to a drop in the TMZ peak current to 0.37 µA, with a corresponding rise in capacitive current for larger suspension volumes.

### 3.3. Influence of Buffer pH

**Figure 7:**
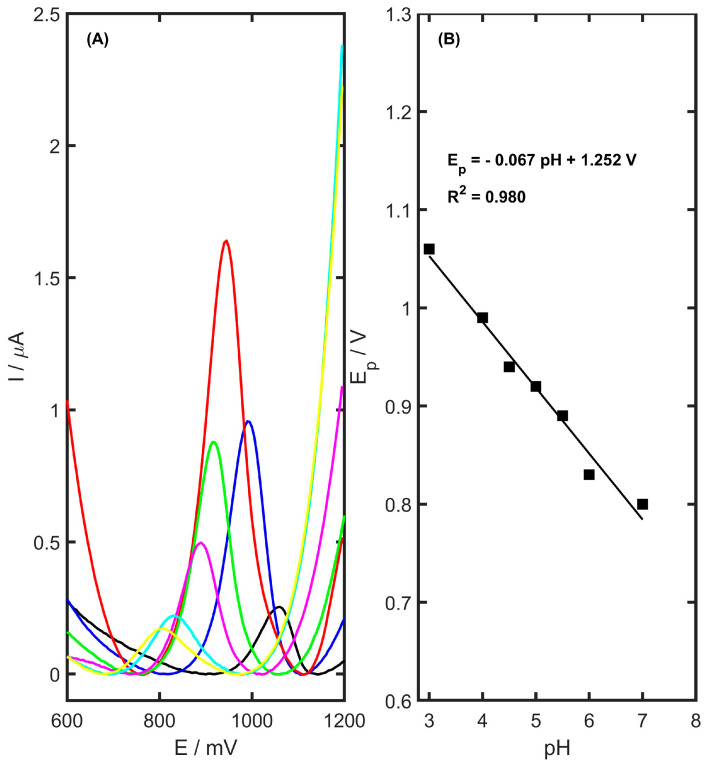
(**A**) DP curves (after background reduction) recorded in various pH levels of acetate buffer for 2 µM TMZ on the YSZTiO_2_-CB/GCE sensor (pH 7.0—yellow, pH 6.0—light blue, pH 5.5—pink, pH 5.0—green, pH 4.5—red, pH 4.0—blue, pH 3.0—black). (**B**) Trimetazidine potential peak dependence on supporting electrolyte pH in the range of 3.0–7.0.

The electrochemical behavior of TMZ is highly dependent on the pH of the supporting electrolyte. As a result, various buffer solutions were tested to identify the most suitable electrolyte for analysis. The effect of buffer pH on TMZ oxidation was examined, focusing on both the peak position and height. Five different supporting electrolytes were evaluated: acetate buffer (0.05, 0.1 and 0.5 M at pH 4.5), phosphate buffer (0.1 M at pH 5.5), KCl (0.1 M at pH 5.0), ammonia buffer (0.1 M at pH 8.2), and borate buffer (0.05 M at pH 9.1). No detectable signal for TMZ was observed in the ammonia buffer. However, TMZ peaks were recorded in the following electrolytes: acetate buffer (0.05 M) with I_p_ = 1.63 ± 0.03 µA and E_p_ = 943 mV; phosphate buffer with I_p_ = 0.33 ± 0.01 µA and E_p_ = 860 mV; KCl with I_p_ = 0.110 ± 0.005 µA and E_p_ = 856 mV; and lastly, borate buffer with I_p_ = 0.150 ± 0.005 µA and E_p_ = 684 mV. The highest current peak was obtained using the acetate buffer. The ionic strength of this buffer was varied with concentrations of 0.05, 0.1, and 0.5 M, and the highest current peak for TMZ was observed at 0.05 M. Additionally, the variation in the TMZ signal was analyzed in relation to the pH of the acetate buffer, tested between pH 3–7. The presented differential pulse voltammograms in Figure 7 show a sharp increase in the anodic peak current from 3.0, peaking at pH 4.5, followed by a significant decrease.

### 3.4. Investigation of the YSZTiO_2_-CB/GC Electrode Mechanism

**Figure 8:**
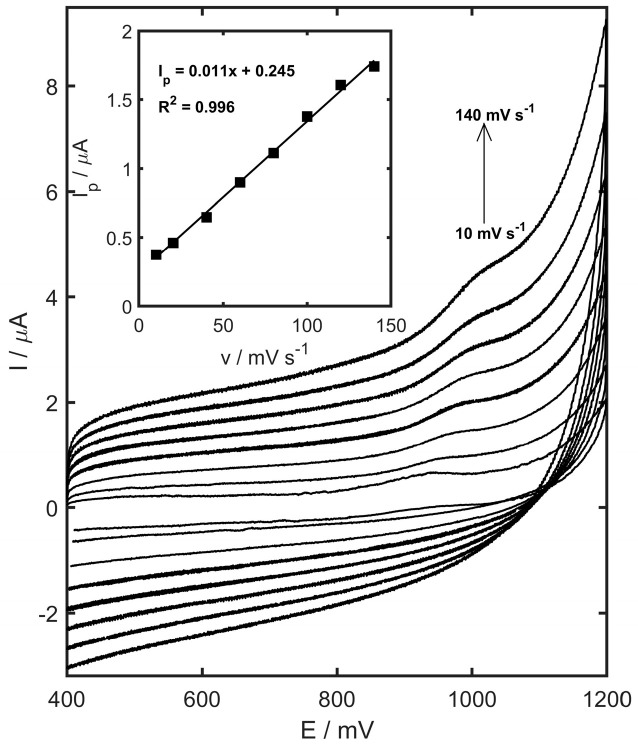
Cyclic voltammograms of 10 µM TMZ in 0.05 M acetate buffer (pH 4.5) measured on the glassy carbon electrode modified with YSZTiO_2_ and carbon black. Scan rate values: 10, 20, 40, 60, 80, 100, 120, and 140 mV s^−1^.

Electrode reactions are generally governed by mechanisms that are either adsorption or diffusion-controlled. To differentiate between these, examining how variations in scan rate (v) influence the peak current (I_p_) offers valuable insights into the dominant process. To gain a better understanding of the mechanism behind TMZ oxidation at the YSZTiO_2_-CB/GCE, cyclic voltammetry measurements were conducted at scan rates ranging from 10 to 140 mV s^−1^ (within the potential range of 400 to 1200 mV, and a TMZ concentration of 10µM). The results (Figure 8) revealed the absence of a reduction peak in the cathodic scan, indicating that the TMZ oxidation process on the YSZTiO_2_-CB/GCE is irreversible. Additionally, a linear relationship was observed between the peak current and the scan rate, implying that the oxidation of trimetazidine follows an adsorption-controlled mechanism.

The number of electrons involved in the oxidation of TMZ can be estimated using the following equation [47]:

(3) $$n=\frac{0.048}{\left|E_{p}-E_{p1/2}\right|}$$

The *αn* value calculated from the equation was equal to 0.92, the charge transfer coefficient (*α*) is assumed to be 0.5, and the number of electrons exchanged during the oxidation reaction is assumed to be ca. 2.

To examine how pH influences the oxidation of TMZ at the developed sensor, cyclic voltammetry (CV) measurements were taken in 0.05 M acetate buffer solutions with pH values ranging from 3.0 to 7.0, using a 2 µM concentration of trimetazidine. Within this pH range, a linear shift in the anodic peak potential towards more negative values was observed as the pH increased (Figure 7B). The relationship between the oxidation peak potential and pH is expressed by the following equation:

(4) $$E_{p}=-0.067\;pH+1.252\;V$$

**Figure 9:**
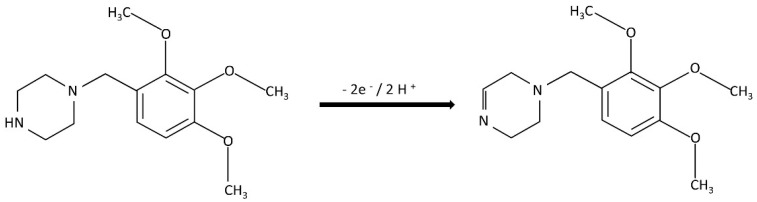
The suggested mechanism for electro-oxidation of TMZ on YSZTiO_2_-CB/GCE.

The slope, which was close to the theoretical value of 0.059 V pH^−1^, suggests that an equal number of exchanged protons and electrons are involved in the oxidation of TMZ at the YSZTiO_2_-CB/GC electrode. Based on these findings and previous studies on the electrooxidation of amine derivatives, it can be proposed that the oxidation mechanism of trimetazidine occurs at the nitrogen atoms of the piperazine rings, involving the removal of two protons following protonation of the secondary amine group. The proposed oxidation mechanism of TMZ on the YSZTiO_2_-CB/GC electrode is depicted in Figure 9. These results align with earlier studies on the oxidation of TMZ at modified electrodes [3,48].

### 3.5. Optimization of Experimental Parameters for Quantitative Analysis

Optimizing the parameters of the DPV technique is crucial for improving sensitivity and detecting low concentrations of TMZ. The optimization process was conducted in 0.05 M acetate buffer (pH 4.5) with the TMZ concentration of 2 µM. To ensure consistent and repeatable TMZ signals, a rest period of 15 s between each voltammogram was maintained. Several experimental parameters were evaluated, including sampling time (t_s_) Ranging from 10 to 50 ms, waiting time (t_w_) from 10 to 50 ms, step potential (E_s_) between 1 and 10 mV, and pulse amplitude (ΔE) from 5 to 100 mV in both positive and negative modes. The optimal conditions were found to be t_s_ = t_w_ = 20 ms, E_s_ = 5 mV, and ΔE = 40 mV. These optimized parameters were used in all subsequent experiments.

### 3.6. Influence of Preconcentration Potential and Time on TMZ Signal

The detection of low TMZ concentrations can be achieved by incorporating a preconcentration step before the anodic scan. Both preconcentration potential and time play critical roles in influencing voltammetry analysis. In this experiment, conducted in 0.05 M acetate buffer (pH 4.5) with 2 µM of TMZ, the preconcentration potential was tested within a range of 0 to 600 mV. It was found that the TMZ peak current was unaffected by the preconcentration potential, and 400 mV was chosen as the optimal value for TMZ detection.

**Figure 10:**
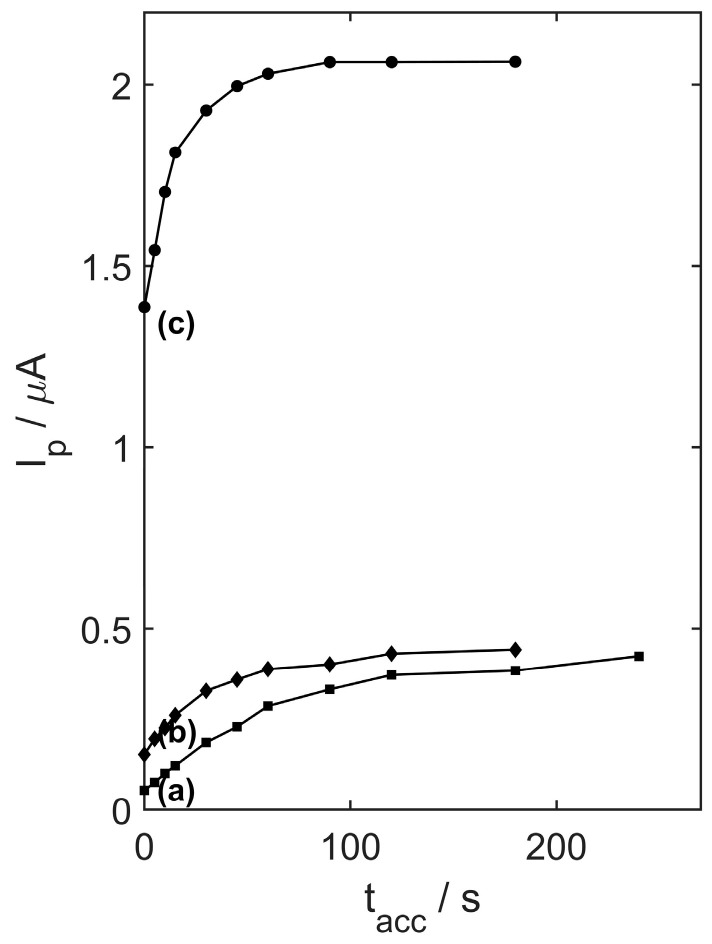
The dependence of TMZ peak current on preconcentration time in the range of 0 to 240 s for (**a**) 0.05 µM, (**b**) 0.2 µM, and (**c**) 2 µM trimetazidine concentration in 0.05 M acetate buffer (pH 4.5).

The effect of preconcentration time was also evaluated under the same experimental conditions. A rest period of 15 s was included between measurements. Preconcentration time was varied from 0 to 240 s, and the relationship between peak current (I_p_) and preconcentration time (t_acc_) is shown in Figure 10. The results indicate that the peak current increased with longer preconcentration times, with the maximum current achieved depending on the concentration of TMZ. For 2 µM of TMZ, the highest current(1.93 µA) was observed att_acc_ = 30 s, whereas for 0.2 µM, the peak current(0.36 µA) occurred at t_acc_ = 45 s. For the lowest concentration (0.05 µM) of TMZ, the peak current (0.33 µA) was reached at 90 s. Based on these findings, a preconcentration time of 0 s was used for 2 µM TMZ, 30 s for 0.2 µM, and 60 s for 0.05 µM in further analyses using the YSZTiO_2_-CB/GCE.

### 3.7. Interference Studies

The capability of the proposed sensor to detect TMZ in real samples, including pharmaceuticals, urine, blood plasma, and digestive juice, was assessed. A range of organic and inorganic substances was examined: Mg(II), Ca(II), K(I) (50 µM added); Cu(II), Pb(II), Zn(II), Mn(II) (5 µM added); SO_4_^2−^, NO^3−^, Cl^−^, CO_3_^2−^, and PO_4_^3−^ (1 mM added). The results demonstrated that the presence of these cations and anions did not significantly affect the TMZ peak current or its potential. However, Mn(II) posed a challenge in interpreting the TMZ signal due to its oxidation peak at around 866 mV, which is close to the TMZ peak at 943 mV. As a result, interference from Mn(II) could not be fully evaluated. In addition, several organic compounds were tested: glucose, saccharose (50 µM added), citric acid (100 µM added), lactose monohydrate, ascorbic acid, urine acid, aspartame, caffeine, acetaminophen, starch, talc (20 µM added each), magnesium stearate, microcrystalline cellulose, titanium dioxide (5 mg per 10 mL of electrolyte), Triton X-100 (2.5 ppm added), saliva, gastric juice, and intestinal juice (20 µL per 10 mL of electrolyte). Only the addition of citric acid caused a decrease in the peak current equal to 18%, and Triton X-100 caused an increase in the peak current equal to 40%.

### 3.8. Analytical Performance

**Figure 11:**
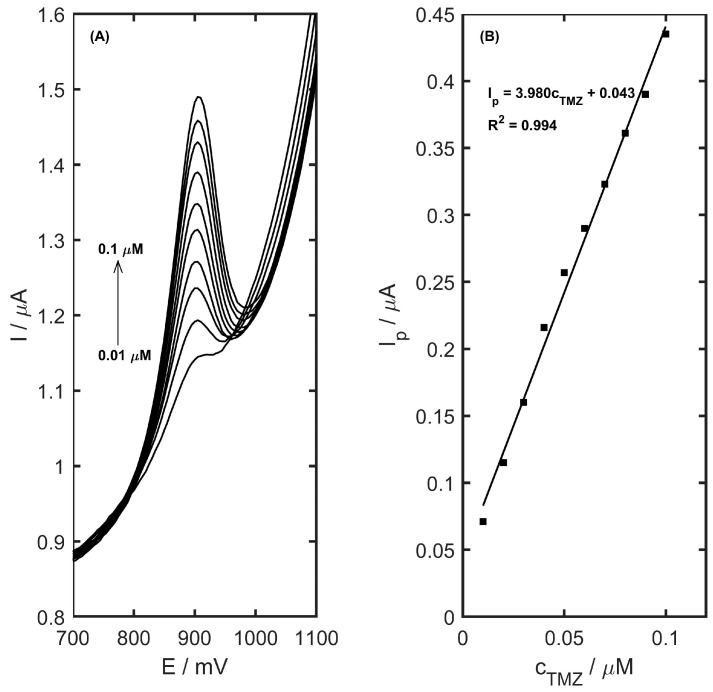
(**A**) DPV calibration voltammograms and (**B**) calibration curves obtained for trimetazidine concentration in the range of 0.01 to 0.1 µM in 0.05 M acetate buffer (pH 4.5), preconcentration time 60 s.

Calibration plays a crucial role in evaluating the analytical performance of the method. The calibration voltammograms, along with the respective calibration plots, are shown in Figure 11. This study was carried out in a 0.05 M acetate buffer solution (pH 4.5) with an accumulation potential of 400 mV. Linearity was observed for trimetazidine concentrations, ranging from 0.01 to 0.1 µM (Figure 11B) (R^2^ = 0.994; slope 3.98 ± 0.12 µA µM^−1^; intercept 0.043 ± 0.007). The detection limit calculated using the equation LOD = 3.3 SD/b (where SD is the standard deviation of current for a blank, and b is the slope of the calibration curve) was determined to be 5.5 nM for a 60 s accumulation period, which is competitive in comparison and is a very good result compared to other electrochemical sensors (Table 3). The calibration curve parameters for TMZ concentrations in the ranges of 0.1–1 µM and 1–7 µM are summarized in Table 4. The limit of quantification (LOQ = 10 SD/b) was computed to be 16.7 nM for the same accumulation time of 60 s. Reproducibility of the electrode was assessed for TMZ concentrations of 2 µM and 0.05 µM, with relative standard deviation (RSD) values of 4.4% and 3.3% (n = 10), respectively. Stability was maintained for up to 300 consecutive measurements on a single electrode and remained functional for up to 1 month.

**Figure 12:**
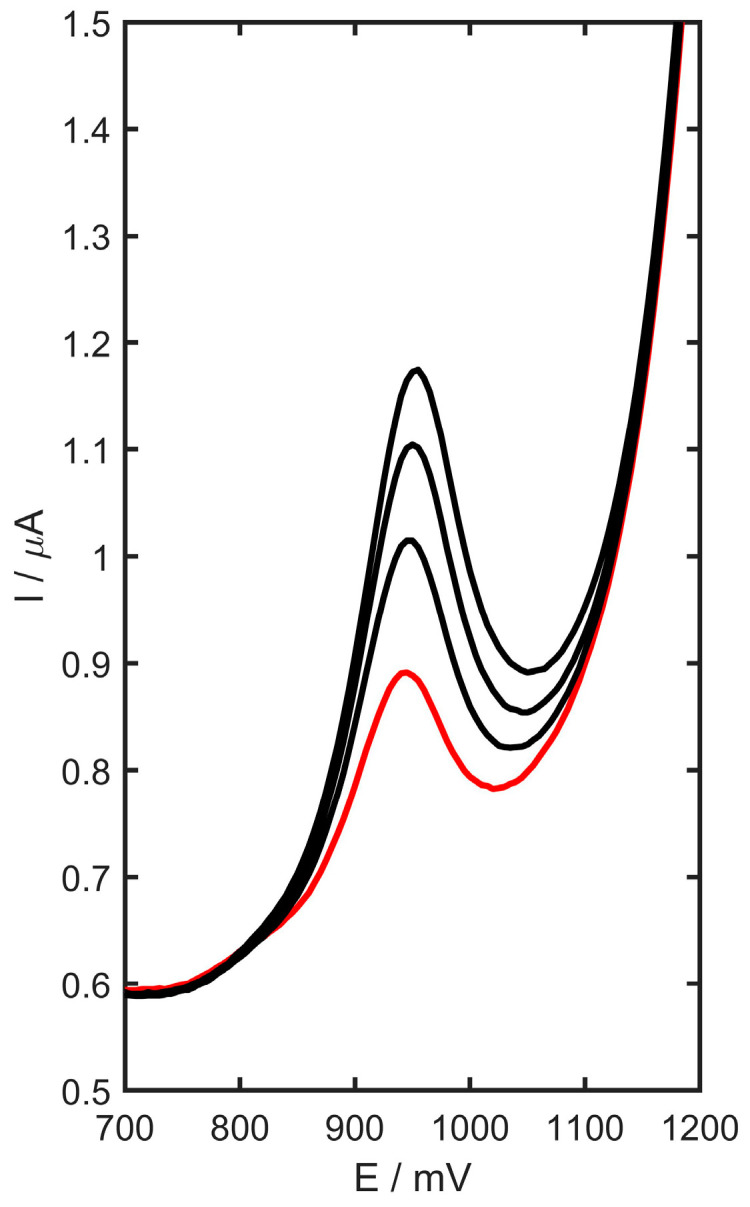
DP voltammograms of trimetazidine determination in human plasma samples (human plasma curve marked as red, three consecutive additions of the standard marked as black).

This electrode design and methodology were applied to the detection of trimetazidine in pharmaceutical tablets, urine as well as blood plasma samples. One pharmaceutical tablet containing trimetazidine was selected for the analysis, with the sample preparation procedure detailed in Section 2.3.3. All measurements utilized the standard addition method and the procedure described in Section 2.3.5. The results, displayed in Table 5, revealed that the mass of trimetazidine per tablet was 34.4 ± 1.8 mg. The received recoveries rates ranged from 97.7 to 102.3%, demonstrating that the yttria-stabilized zirconia doped with titanium oxide—carbon black—modified GC electrode is suitable for the determination of TMZ in various sample types. The DP voltammogram for the blood plasma sample with standard additions of TMZ is presented in Figure 12.

### 3.9. Extraction into Artificial Digestive Juices

**Figure 13:**
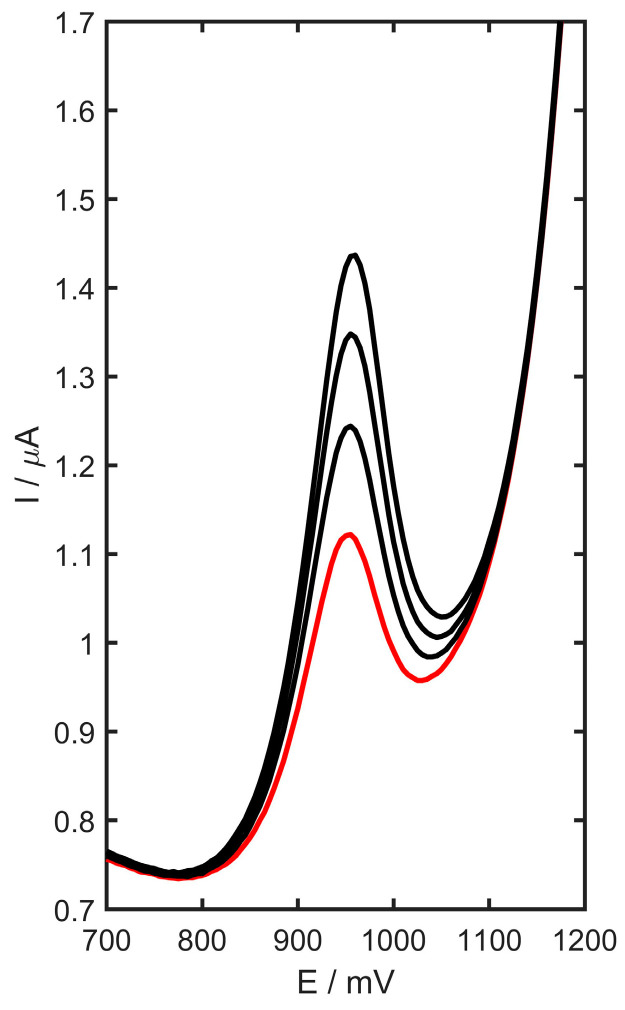
DP voltammograms of trimetazidine determination in 5 µL gastric juice solution after 120 min of extraction (gastric juice curve marked as red, three consecutive additions of the standard marked as black).

As an additional illustration confirming the applicability of the method, an experiment was conducted to evaluate trimetazidine extraction into the digestive system. The glassy carbon electrode was modified with YSZTiO_2,_ and carbon black was applied to detect the active substances in artificial digestive fluids (both gastric and intestinal). One or two pharmaceutical tablets were dissolved in artificial digestive juice, and the resulting extracts were analyzed using the standard addition method, following the procedure outlined in Section 2.3.4. The DP voltammogram for the trimetazidine with standard additions is shown in Figure 13. The extraction efficiency for trimetazidine extraction into gastric juice or intestinal juice is summarized in Table 6.

The highest extraction efficiency was recorded when two tablets were used, yielding 52.8 and 55.6% in intestinal juice solution. Moreover, the extraction efficiency was higher in the intestinal juice compared to gastric juice. This suggests that the extraction process is more effective in the intestinal, which can be attributed to the slightly alkaline pH (around pH 8) of the intestinal juice.

## 4. Conclusions

This study presents a highly sensitive voltammetric approach for determining trimetazidine (TMZ). For the first time, a new electrochemical biosensor was employed, based on a glassy carbon electrode (GCE) modified with yttria-stabilized zirconia doped with titanium oxide (YSZTiO_2_) and carbon black (CB). The developed sensor offers several advantages, including simplicity of the fabrication process, cost-effectiveness, and high reproducibility. The modification significantly enhanced the sensitivity, with improvements of up to 2-fold compared to the carbon black–modified glassy carbon electrode and 20-fold compared to the bare glassy carbon electrode. Measurement parameters, such as the supporting electrolyte and instrumental conditions, were optimized for the best performance. Additionally, the effects of potential interfering substances on the trimetazidine signal were investigated. The limit of detection (LOD) and quantification (LOQ) were calculated, with values of 5.5 nM and 16.7 nM, respectively, for a preconcentration time of 60 s. These results represent an improvement compared to other electrochemical sensors for TMZ determination reported in the literature. The proposed sensor was successfully applied to the analysis of TMZ in pharmaceutical samples, as well as in urine and human plasma. The recovery rates, ranging between 97.7 and 102.3%, confirm the effectiveness of the zirconia doped with yttria and titanium dioxide–carbon black modified glassy carbon electrode for detecting trimetazidine in real samples. Additionally, trimetazidine extraction was tested in artificial digestive juices, resulting showing significant release in intestinal fluid. The proposed sensor demonstrated the ability to detect TMZ in both gastric and intestinal juices.

In summary, the developed voltammetric sensor, featuring YSZTiO_2_ nanopowder, carbon black modified glassy carbon electrode, has proven to be highly versatile and suitable for use in applicability in pharmaceuticals, biological fluids, and digestive systems. This voltammetric method shows considerable promise for routine TMZ determination in stationary conditions.

## Figures and Tables

**Table 1:** The electrochemical parameters obtained for the GC electrode modified with layers of YSZTiO_2_, carbon black, and a combined carbon black with YSZTiO_2_ coating, using a scan rate of 100 mV s^−1^.

	E_pa_, mV	E_pc_, mV	ΔE_p_, mV	I_pa_, µA	I_pc_, µA	I_pa_/I_pc_	A, cm^3^
GC	294	226	68	11.59	12.07	0.96	0.050
YSZTiO_2_/GC	291	231	60	15.12	15.73	0.96	0.065
CB/GC	291	232	59	16.81	16.41	1.03	0.073
YSZTiO_2_-CB/GC	297	237	60	20.68	20.47	1.01	0.088

**Table 2:** The comparison of the parameters and charge-transfer-resistance values derived from EIS measurements for the modified and unmodified electrodes.

	GCE	YSZTiO_2_/GCE	CB/GCE	YSZTiO_2_-CB/GCE
CPE [µF]	2.97	0.82	2.97	3.66
*R*_2_ [Ω]	20.5	15.6	16.1	18.0
*R_ct_*, [Ω]	56.1	409	381	159
*W*_1_ [kΩ]	130.4	126.4	90.3	127.4

**Table 3:** Comparison of different developed sensors used in the determination of TMZ.

Electrode	Technique	Electrolytic Solution	Linear Range, µM	LOD, nM	Reference
GCE	SWV	Acetate buffer pH 5.0	0.5–5.0	75.3	[49]
CdS/MOF-74T/GCE	SWV	Acetate buffer pH 4.0	0.05–220	27.4	[50]
PACPE	CV	B-R buffer solution pH 4.0	0.5–50	150	[51]
GCE/PEDOT/ACN	SWV	Phosphate buffer pH 3.0	0.1–100	12.0	[3]
YSZTiO_2_-CB/GCE	DPV	Acetate buffer pH 4.5	0.01–0.1	5.5	This work

GCE—glassy carbon electrode; CdS/MOF-74T/GCE—CdS and metal–organic frameworks glassy carbon electrode, PACPE—pre-anodized carbon paste electrode, GCE/PEDOT/CAN—Poly(3,4-ethylenedioxythiophene)-coated glassy carbon electrode.

**Table 4:** Characteristics of the calibration plots of trimetazidine.

Preconcentration Time (s)	Linearity Range (µM)	Equation	Correlation Coefficient	Detection Limit (nM)
0	1–7	y = 0.291x + 0.077	0.999	210
30	0.1–1	y = 2.339x + 0.088	0.998	17.8
60	0.01–0.1	y = 3.980x + 0.043	0.997	5.5

**Table 5:** Trimetazidine concentrations and calculated recoveries in pharmaceutical products, urine, and blood plasma samples.

Sample	Added, µM	Found, µM	Recovery, %
Tablet	0	1.01 ± 0.04	-
	1	2.00 ± 0.06	97.7
	2	2.99 ± 0.05	97.9
	3	4.01 ± 0.09	100.9
Urine diluted 200×	0	ND	-
	1	1.02 ± 0.09	102.3
	2	2.03 ± 0.01	100.8
	3	3.01 ± 0.04	97.9
Plasma diluted 100×	0	ND	-
	1	0.99 ± 0.02	98.7
	2	1.99 ± 0.15	99.9
	3	2.98 ± 0.03	99.4

**Table 6:** The efficiency of trimetazidine extraction into gastric juice or intestinal juice. The extraction time into gastric juice is 60 or 120 min and into intestinal juice is 150 min.

Digestive Juice	Tablet	Extraction Efficiency [%]	
Time of extraction [min]		60 min	120 min
Gastric juice	1	21.9 ± 1.0	17.8 ± 7.8
	2	17.1 ± 0.6	18.2 ± 0.9
Intestinal juice	1	36.1 ± 2.4	33.3 ± 1.7
	2	52.8 ± 3.3	55.6 ± 8.3

## Data Availability

The data presented in this study are available upon request from the corresponding author. The data are not publicly available due to the excessive amount of data in the repository.
